# Clinical learning experiences of male nursing students in a Bachelor of Nursing programme: Strategies to overcome challenges

**DOI:** 10.4102/curationis.v38i2.1517

**Published:** 2015-09-30

**Authors:** Sibusiso F. Buthelezi, Lorrain P. Fakude, Penny D. Martin, Felicity M. Daniels

**Affiliations:** 1 School of Nursing, University of the Western Cape, South Africa

## Abstract

**Background:**

Male nursing students are faced with more challenges in the clinical setting than their female counterparts. The ways in which male nurses are viewed and received by nursing staff and patients have an impact on how they perceive themselves and their role in the profession. These perceptions of self have a significant impact on their self-esteem. This study was conducted to explore the clinical learning experiences of male nursing students at a university during their placement in clinical settings in the Western Cape Province, and how these experiences impacted on their self-esteem.

**Objectives:**

To describe the learning experiences of male nursing students during placement in clinical settings, and how these impact on their self-esteem.

**Method:**

A qualitative, exploratory study was conducted. Purposive sampling was used to select participants. Three focus group (FG) discussions, consisting of six participants per group, were used to collect data. Data analysis was conducted by means of Coliazzi's (1978) seven steps method of qualitative analysis.

**Study findings:**

The following three major themes were identified: experiences that related to the constraints in the learning environment, the impact on the self-esteem, and the social support of students working in a female-dominated profession.

**Conclusion:**

Male nurses should be supported in nursing training, as the rate at which males enter the profession is increasing.

## Introduction

Despite the global increase in the number of males entering the nursing profession (Eswi & El Sayed 2011:94), male nurses remain a minority group (Lerardi, Fitzgerald & Holland 2010:215; Meadus & Twomey 2011:269). In the United State of America (USA) male nurses constituted 9.6% in 2011 (U.S. Census Bureau 2013:2), in Canada 5.8%, and in China less than 1% of the total population of nurses (Rajacich et al. 2013:71; Wang et al. 2011:36). In South Africa, as in many other countries, nursing remains a female-dominated profession.

It is evident that although nursing has traditionally been a female-dominated profession (Meadus & Twomey 2011:269), there have been a small number of males entering the profession. At the University of the Western Cape (UWC) the intake of students into the nursing faculty increased from 120 to 300 in 2004 (Jeggels, Traut & Kwast 2010:52). Globally males have also played a role in nursing and caring for patients and being employed in the health care sectors (Ozdemir, Akansel & Tunk 2008:154). Some of these roles include, but are not limited, to males of various religious orders providing nursing care and protection to the sick, wounded and dying in the time of war and peace (Evans 2004:322). Evans (2004:321) states that the failure to recognise men's contribution in nursing leaves male nurses with very little information about their professional background. Smith (2006:263) and Wilson (2005:222) allude to the challenges males encounter in their work as nurses. These include questions about their masculinity or sexuality, discrimination because of their gender, the absence of suitable role models, lack of support, feelings of isolation, poor instruction on the appropriate use of touch and unequal clinical opportunities. These challenges may affect males who choose to register for education and training in nursing at various institutions of learning.

The clinical learning environment (CLE) is defined as an ‘interactive network of forces within the clinical setting which influence the students’ clinical learning outcomes’ (Chan 2002:581; Dunn & Hansford 1997:1299). The CLE provides real life situations and allows the student nurse to use cognitive, psycho-motor and affective skills, which are vital for the development of the specific knowledge, problem-solving skills, and values required in the nursing profession (Kapucu & Bulut 2011:1149; Salamonson et al. 2011:2669).

## Problem statement

Important growth points for students in the CLE are professional and self-development. A non-conducive environment may not only impact on the students’ ability to learn clinical skills but also on their self-esteem which will affect both self and professional maturity. In a school of nursing, anecdotal concerns were raised by male nursing students to their clinical supervisor about their clinical learning experience in caring for females in a female-dominated profession. This prompted a need to deeply understand the lived experiences of learner student males regarding their clinical learning in nursing practice. The research questions posed were: what are the lived experiences of male student nurses during their placement in the clinical settings, and how these impact on their self-esteem? Whilst studies have been carried out elsewhere on male student nurses’ experiences in the clinical setting, there is a paucity of literature within the South African context given the small number of males studying nursing as a profession. The UWC, being the largest enrolling university for the undergraduate nursing programme, provided fertile ground for the researcher to explore and describe male student nurses’ clinical learning experiences in nursing practice.

## Purpose of the study

The purpose of this study was to explore the clinical learning experience of male student nurses during placement in the clinical setting, and how this impact on their self-esteem.

## Methodology

### Setting

The study was conducted at a school of nursing in the Western Cape Province, with more than 1000 undergraduates with a preceptor: student ratio of 1:30. The skills laboratory method is used as the preferred teaching method in clinical practice. Male nursing students comprise around 8% of the student population enrolled for a four-year degree programme.

The school places all the undergraduate nursing students in accredited clinical settings which consist of hospitals and community health care centres (CHC). In the first year of study, students are taught foundational knowledge about nursing. Placements in a clinical setting are limited as the focus is on theory. However, in the second year of study, the nursing programme focuses on the acquisition of knowledge, skills and attitudes in general nursing science. Students work in a variety of wards to acquire the necessary skills and knowledge in order to care for patients. In the third year of the nursing programme, the focus is on midwifery, community and child health. Students work for three days per week in these disciplines on a rotational basis in order to meet the South African Nursing Council's (SANC) clinical requirements. In the fourth year the focus is on psychiatric nursing science. Students work in the mental health care settings for three days per week in order to acquire skills, knowledge and the correct attitude to care for people who are mentally challenged and intellectually disabled.

### Design

A qualitative research approach using focus groups (FGs) was followed in this study, as little was known about the phenomenon of male learner nurses placed in clinical settings. The researcher wanted to identify, explore, and describe the phenomenon. A descriptive phenomenological design was adopted in the study to investigate the lived experiences of male nursing students based on the descriptions that they could provide. According to Streubert-Speziale and Carpenter (2007:76), descriptive phenomenology involves direct exploration, analysis, and description of a phenomenon. It is carried out as freely as possible and without any presumptions whilst maximising the intuitive presentation of the participants’ experiences.

### Participants

The accessible population was 107 male nursing students enrolled for a nursing programme at a university for their second (*n* = 42), third (*n* = 33) and fourth (*n* = 32) years of study. They were considered to be knowledgeable and to possess sufficient experience regarding clinical learning during placement in the clinical setting. Male students registered in the first two years of the foundation five-year programme and male students who are registered at first-year level in a four-year nursing programme were excluded because they do not have sufficient clinical learning experience at this level of the programme. Purposive sampling was used to select 18 participants for the study. As the intention was to conduct one FG for each year level of study, the first six students to volunteer from different cultures per yearlevel were approached to participate in the study. This method of sampling was chosen to constitute the group of participants from different year levels and also to enable the researcher in the setting of the study to mix the group of participants from different cultures in the country. Purposive sampling allowed the researcher to generate appropriate, information-rich, and useful data from the participants (Green & Thorogood 2014:121). The inclusion criteria were voluntary participation. Written informed consent forms were signed after the researcher explained the benefits of the study to the participants.

### Material and methods

Three FGs (one for each year of study) were conducted to collect the data. Each group had six participants. Focus group is an interaction between six to eight pre-selected participants who focus on a specific issue whilst being led by a researcher (De Vos et al. 2011:362–365). As each group was a homogenous group (in terms of gender and the fact that they were nursing students) and all members were sharing particular experiences from their year of study, the configuration enabled the participants to interact freely, and also made the environment safer to share the information about the phenomenon under investigation (Burns & Grove 2009:513; Green & Thorogood 2014:143).

### Data collection

The researcher initially asked one open-ended question followed by numerous probing questions to obtain in-depth responses from the participants. The data were collected by the researcher and his assistant. The assistant researcher's role was to take comprehensive notes because the researcher had to focus on facilitating the discussion, encouraging participation, and recognising key ideas. The audio recorder was introduced to the participants as a tool that would assist the researcher with recording the information discussed by participants without omitting any information. Each FG interview lasted between 45 and 60 minutes. These interviews were conducted until no new information was emerging, that is, to saturation point. All interviews were conducted in English and were transcribed verbatim.

To ensure rigor of data collection, the four criteria for trustworthiness as set out by Lincoln and Guba (1985) in Polit and Beck (2012:584) were used. *Credibility* was ensured by member checking, that is, sending the transcripts back to the participants to confirm that what was transcribed reflected what they had said during the FG interviews. Credibility was further ensured by obeying the principles of a phenomenological method; the researcher bracketed his own experiences and kept the field notes. All transcripts of the interviews were given to an independent coder for coding (Creswell 2013:333). *Dependability* was ensured by keeping an audit trail of the research process. Independent verification of coding by an independent coder also enhanced the dependability of the study. *Confirmability* was ensured by creating an audit trail to determine whether the conclusions, interpretations, and recommendations could be traced to the source. Actual quotations from the participants were also used in the written report of the study. Lastly, *transferability* was ensured by the presentation of a detailed description of the participants, research context, and setting supported by appropriate quotations.

### Data analysis

Coliazzi's (1978) method of qualitative analysis was employed to analyse the data. This method required the researcher to read transcripts several times with the purpose of gaining a general impression of their content. Important phrases pertaining to the experience of clinical learning were identified. The meaning was formulated from the significant phrases and these meanings formed the basis of the categories. The categories were then organised in order to arrive at a thematic description that answered the research question. Every category and theme was substantiated by quotations from the raw data. These were then compared and contrasted with existing relevant literature and research.

## Ethical considerations

### Information removed to ensure blind peer review

#### Results

Three main themes with sub-themes and categories emerged from the data in respect of the experiences of male nursing students who were working in a CLE, namely constraints in the clinical learning process, the impact on the students’ self-esteem, and social support. This article focuses on the theme around the self-esteem (Table 1).

**TABLE 1:** Themes.

Theme	The impact on the students’ self-esteem
Decreased sense of selfworth	Students experienced nursing as a female-dominated profession which resulted in a lack of a sense of belonging.
	Students felt devalued because of the verbal abuse from clinical supervisors.
Influence on self-esteem	Students gained self-confidence as they matured in the profession.
	Students felt rejected and distrusted by female patients in the clinical setting.
	Students experienced feelings of discomfort whilst caring for female patients.
	Students were motivated and assured by the presence of male nurses in the clinical setting.
The influence of cultural beliefs	Cultural background influenced students’ beliefs and behaviours in caring for female patients.

#### Demographic information

A total of 18 male student nurses participated in the FG discussion. Most (*n* = 16) of the participants were between the ages of 20 and 26. Two participants were between the ages of 36 and 39. Most of the participants were black African (*n* = 13). Mixed race were five (*n* = 5). All participants were able to speak English. However, their mother tongue was Afrikaans, SeTswana, IsiXhosa and IsiZulu.

Students experienced the CLE as having an impact on the self. The impact related to a decreased sense of self-worth, affecting the self-esteem and of the influence of cultural beliefs.

#### Sub-theme: Decreased sense of self-worth

Students experienced nursing as a profession which was dominated by females because they did not see many males working in the clinical setting. A participant stated: ‘I was just seeing nursing as like a profession whereby there is no male …’ (FG2, P3). Furthermore, they reported that they felt that they did not belong in a female-dominated profession. A student reported:

‘… [*Y*]ou think that this is not the world (profession) for you …’ (FG3, P6)

‘… [*A*]ll these problems we face them because now you are going to the female dominated world (female dominated profession) and nobody even told us (male students) that there is challenges in this female dominated world …’ (FG3, P1)

Meyer (2012:76) found that male nursing students felt that they did not belong in midwifery because there were no notable male role models.

This finding is supported by Moagi, Van Rensburg and Maritz (2013:54) in their study conducted in Pretoria, South Africa, which revealed that male nursing students experience isolation which may be derived from being male in a predominantly female career. Another participant re-affirmed the female dominance when he reported ‘… we can’t deny that this profession is dominated by women …’ (FG1, P3).

Other participants described their experience of nursing by alluding to the fact that there are only a limited number of males in the nursing profession, as is illustrated by the following quotations:

‘… [*I*]t's been three years now, I cannot say … I cannot mention more than five people that I have work with who are male nurses. Everywhere I go females are dominating …’ (FG2, P4)

‘… you don’t see male nurses in the ward …’ (FG3, P5)

and:

‘… I also didn’t see a lot of male students …’ (FG1, P2)

In Canada, a study conducted by Meadus and Twomey (2011:257) found that male students felt like intruders in the nursing profession. Tzeng et al. (2009:1) revealed that male students faced more gender based role stress than their female counterparts. In addition, students felt devalued and ashamed by the clinical supervisors’ negative behaviour which was described by a student as: ‘… I’m [*male student*] not a good person…’ (FG1, P5) Another student reported that:

‘The ward was full of patients, the supervisor shouted me … from that day in that cubicle the patients were like I’m [*male student*] not a good person, I know nothing about medication because my supervisor shouted me … Sometimes the treatment of supervisors it's really, really, not good …’ (FG1, P1)

Other participants indicated that they could not tolerate the verbal abuse and perceived it as chastisement, likening it to being: ‘… shouted like small children …’ (FG3, P1), ‘… I cannot take a shouting, because we are being shouted. We are being shouted like small children in hospital …’ (FG3, P3), and: ‘… to add on what he is saying; one thing I can say about supervisor X she is rude if she wants to, she is rude when she wants to …’ (FG2, P5).

Mabuda, Potgieter and Alberts (2008:22–23) reported that students were not supported by the ward sisters and were often scolded in front of the patients and the ward sisters’ colleagues. This phenomenon could also be experienced by female students, but it came out strongly from the male students who participated in this study. A comparative study conducted by Mabrouk Abd El Rahman (2014:10) on the perception of student nurses’ bullying behaviours and coping strategies used in clinical settings, found that male students reported being bullied significantly by nursing staff in a clinical environment and subjected to negative remarks. This phenomenon must not be neglected or taken lightly even when it happens to male students. Acknowledging it would help in breaking the cycle of ill-treating students in the clinical environment, which affect their self-esteem negatively.

These students may have felt humiliated because they felt that they were not being recognised as adults, which is important in the South African context, especially for black men who are raised in a culture that says that a woman does not shout at a man under any circumstances.

#### Sub-theme: Influence on self-esteem

Students appeared to feel more vulnerable in the clinical setting during the first few years of the education and training as a nurse. However, when they reached the fourth year of study, they matured, gained self-confidence and may have learnt more about how to deal with people. Therefore, some of the fourth year students did not feel intimidated by the clinical supervisors. The following quotation illustrates this:

‘… [*T*]hey [*clinical supervisor*] have to be friendly to the fourth year, maybe it's because the students have learned and they have this academic knowledge, now a person [*clinical supervisor*] cannot intimidate them [*students*].’ (FG3, P4)

Similar findings were reported in a study conducted in South Africa by Tshabalala (2011:33) on the experiences of a group of student nurses regarding mentoring during clinical practice. The study found that students in their fourth year of study were more independent and confident than when they had been in their first year. This also sounds like a finding that is not unique to the male students

Participants verbalised that they experienced rejection, particularly from female patients. In addition, participants felt that female patients did not trust them to provide nursing care in the same way as female nurses can do. This might have been an emotional situation that students were faced with, because being rejected and not to be trusted because of being a male could made them feel that they are not being accepted by patients. This may have increased the likelihood of them not fully participating in nursing activities, as is illustrated by the following quotations:

‘… [*O*]ur [*male students*] practice is hindered in a way that some patients do not trust us as males because they are not expecting us to do what the female nurses do …’ (FG1, P6)

‘… [*F*]or some patients they don’t even trusts you [*male student nurse*] to give their babies injectable immunisation … they [*patients*] even ask you, can’t you let your colleague, which is a female to do what you want to do …’ (FG2, P2).

‘… [*P*]atients say, no, I cannot be help by male … so, ah … that's one of the things that we face as male in clinical field …’ (FG1, P4).

A study conducted by Eswi and El Sayed (2011:97), exploring the learning experience of Egyptian male student nurses during maternity nursing clinical course, also reported that patients did not trust male students when receiving care from them.

The distrust and rejection by female patients does not only affect the self, but it may also limit opportunities for male students to practise nursing skills and to become competent nurses. As much as the male students were faced with rejection in the clinical setting, it was noted that this was worse when male students were placed in midwifery. This could be because intimate care happens more in midwifery than in other disciplines of nursing. This poses a serious challenge for males, especially because it is compulsory for them to rotate in midwifery. This is where most of the males experienced rejection. The following quotations illustrate this:

‘… [*W*]hile I was doing midwifery I had some challenges … patients who prefer females over males …’ (FG3, P2)

‘… [*W*]hen it comes to pv [*examination per vaginal*] the patient just say: wait! wait! wait! What are you doing? … No! … call somebody else maybe a sister [*female registered nurse*] …’ (FG2, P6)

‘… [*O*]ne of the patient told me no! no! no! I’ve never been touched by a male since I’d been in hospital …’ (FG3, P3)

‘… [*E*]ven in general hospital some female refuse you [*male*] and want a female colleague …’ (FG2, P1)

These findings concur with the findings of Meyer (2012:66) who conducted a study on the experiences of male nurses in midwifery clinical training at a regional hospital in the Eastern Cape. The study found that participants reported that many female patients showed a preference for a female midwifery service provider by refusing treatment that was offered by males. In addition, participants themselves verbalised the discomfort that they experienced whilst caring for female patients. The following quotations illustrate this:

‘I helped that lady [*female patient*] but after that I … told the sister … I don’t think I feel comfortable.’ (FG3, P5)

and:

‘Midwifery … was a hard thing for me. I took time to finish my babies [*birth delivery*]. I took time to finish my pv's [*vaginal examinations*] you know, really I was not comfortable.’ (FG2, P4)

Another study conducted in Australia by Inoue, Chapman and Wynaden (2006:564) revealed that male students found the experience of providing intimate care for women patient challenging. Meyer (2012:68) found that males were not comfortable to sit in a chair in front of a woman's private part to suture the perineum.

Participants, however, felt motivated by the presence of other male nurses which gave them strength to carry on despite all the challenges that they faced in a female-dominated profession. The following quotations illustrate this:

‘… [*I*]t's much easier to relate to … to a male professional nurse than with the female professional nurse. It is really also to see the man [*male nurse*] doing this thing that you are supposed to be doing, it gives you that little bit of a push to do it …’ (FG3, P5).

‘… [*W*]hen I’m around a male [*nursing staff*] from my side I thought when he's doing something I thought ok males can do this, then I join in and do it.’ (FG1, P2)

The presence of male nurses in the clinical setting helped students to feel part of the team and to become comfortable within the nursing team. This increased the level of students’ participation in learning in the clinical environment. The following quotations illustrate this:

‘I would say for me learning in the clinical facilities around other male practitioners makes things to be much more appreciated …’ (FG2, P3)

and:

‘I find it very comfortable even though in my whole years of practice, I met with very few male professionals in the clinical facilities.’ (FG3, P6)

The presence of other males in nursing helped students to cope with the fact that nursing is a profession that is dominated by females. Another student said:

‘… what was nice there was a senior male sister that welcomed us and orientated us, so that really help me a lot to accept the situation …’ (FG1, P5)

In Iran, a study conducted by Zamanzadeh et al. (2013:54) states that male students expressed their desire to interact more often with male role models.

This was also confirmed by Meadus and Twomey (2011:275) who quote a participant as having stated: ‘I was the only guy on the floor period, so I found it much better when there was another male student.’

#### Sub-theme: The influence of cultural beliefs

Cultural background influenced students’ beliefs and behaviours in caring for female patients. Culture still plays a major role in South African communities. This was noted when the students found themselves applying the principles of their culture inproviding care to patients, particularly to females. Students stated:

‘I had difficulties, like since we are from … like different cultures … there's this thing where they say you can’t … you are not allowed to … to see … adults private parts. So … when they say I must wash the old person, I was like hey … it was a female. It was my first time …’ (FG3, P6)

‘I agree … you know sometimes is difficult to work with females who in labour ward … I think it's a cultural thing. Men are not supposed to be there …’ (FG2, P1)

‘I couldn’t wait to finish midwifery just because of that …! Seeing women private parts yoh! It was not good …’ (FG2, P6)

Mxoli (2007), as cited in Meyer (2012:82), reported that one of the pregnant Xhosa participants said, ‘We as Xhosa women are not used to undress in front of a male’. This finding confirms why the males, particularly black students, were concerned about seeing the private body parts of a woman.

## Recommendations

The male students are most affected by a situation in the wards where there are no male patients, and where the clinical skill can be performed only on a female patient. When students are placed, this should be borne in mind.

A positive relationship between the students and the clinical supervisor could have a positive impact on the self. The fact that the nursing profession is dominated by females should not create an environment in which males feel that they do not belong in the profession. More male professional nurses should take the lead to motivate their younger nurses.

The participants’ learning should not be compromised and tasks should not be allocated according to gender. Males should participate in all nursing activities related to the learning objectives which have to be achieved. It needs to be acknowledged that male and female student nurses need to acquire the same knowledge and skills; so there should not be a division of labour on the basis of gender.

Cultural background is a reality; however males should be appropriately orientated to provide care to patients, particularly female patients. Male students do need support in the clinical setting in order to overcome situations that prevent them from fully participating in the nursing care.

In-depth orientation, particularly about the issues of gender in nursing as a female-dominated profession is essential. This orientation should preferably be carried out by a male clinical supervisor or a lecturer. This will ensure that students are fully prepared psychologically and emotionally to deal with the challenges they will face in a clinical setting.

Educators, professional nurses, and clinical supervisors should be aware of gender differences and need to make an effort to provide a teaching and learning environment that is neutral in terms of its gender expectations (Stott 2004:329). Attempts need to be made to allocate tasks without basing it on gender differences. Learning opportunities should be equally allocated to students irrespective of their gender. Professional and practising nurses should allow students to practise nursing skills according to their learning objectives.

High school learners should be exposed to the idea that males are part of this profession and they should be allowed to ask pertinent questions; especially to dispel the stereotype that this profession is only for females, or that males joining the profession are perceived to be less manly.

Workshops could provide opportunities for discussing challenges and concerns with male students as they are more likely to experience unique challenges, and for discussing problems during the stages of their training that would benefit them in terms of an opportunity to exchange their experiences and coping strategies. Using FGs as a medium of supportive exchange is a positive practical recommendation.

It should be acknowledge that peer support is an invaluable building block for the emotional well-being of students and the provision of opportunities for peer mentoring.

## Conclusion

Although there seems to be limited differences between male and female experiences in the literature, as most of the findings relate to nursing students in general (Levett-Jones et al. 2008; Mabuda et al. 2008; Pitkäjärvi et al. 2012), this study illustrates that male nursing students are still experiencing additional challenges in a clinical setting compared to their female counterparts. More support structures for male students in the early years of the training programme should be provided. This will assist them with being more comfortable to share their concerns and build confidence whilst they are becoming professionally more mature.
